# Stop codon readthrough generates a C-terminally extended variant of the human vitamin D receptor with reduced calcitriol response

**DOI:** 10.1074/jbc.M117.818526

**Published:** 2018-01-31

**Authors:** Gary Loughran, Irwin Jungreis, Ioanna Tzani, Michael Power, Ruslan I. Dmitriev, Ivaylo P. Ivanov, Manolis Kellis, John F. Atkins

**Affiliations:** From the ‡ School of Biochemistry and Cell Biology, University College Cork, Cork, Ireland,; § Computer Science and Artificial Intelligence Laboratory (CSAIL), Massachusetts Institute of Technology, Cambridge, Massachusetts 02139-4307, and; ¶ Department of Human Genetics, University of Utah, Salt Lake City, Utah 84112-5330

**Keywords:** nuclear receptor, transcription factor, transfer RNA (tRNA), translation release factor, vitamin D, VDR, calcitriol, PhyloCSF, readthrough, stop codon

## Abstract

Although stop codon readthrough is used extensively by viruses to expand their gene expression, verified instances of mammalian readthrough have only recently been uncovered by systems biology and comparative genomics approaches. Previously, our analysis of conserved protein coding signatures that extend beyond annotated stop codons predicted stop codon readthrough of several mammalian genes, all of which have been validated experimentally. Four mRNAs display highly efficient stop codon readthrough, and these mRNAs have a UGA stop codon immediately followed by CUAG (UGA_CUAG) that is conserved throughout vertebrates. Extending on the identification of this readthrough motif, we here investigated stop codon readthrough, using tissue culture reporter assays, for all previously untested human genes containing UGA_CUAG. The readthrough efficiency of the annotated stop codon for the sequence encoding vitamin D receptor (VDR) was 6.7%. It was the highest of those tested but all showed notable levels of readthrough. The VDR is a member of the nuclear receptor superfamily of ligand-inducible transcription factors, and it binds its major ligand, calcitriol, via its C-terminal ligand-binding domain. Readthrough of the annotated *VDR* mRNA results in a 67 amino acid–long C-terminal extension that generates a VDR proteoform named VDRx. VDRx may form homodimers and heterodimers with VDR but, compared with VDR, VDRx displayed a reduced transcriptional response to calcitriol even in the presence of its partner retinoid X receptor.

## Introduction

Context-dependent codon meaning enriches gene expression. Depending on the nature of relevant context features, the efficiency of specification of the alternative meaning can be set at widely different levels or be subject to regulatory influences. The majority of known occurrences of such dynamic redefinition of codon meaning involve UGA and UAG. Because, in the nearly universal genetic code, these codons usually specify translation termination, specification of an alternative meaning generally involves tRNA competition with release factor for their reading in the ribosomal A-site. In what is commonly termed stop codon readthrough, a near-cognate tRNA performs the decoding with utility deriving from a proportion of the product having a C-terminal extension with an additional function. In these instances, the identity of the amino acid specified by the UGA or UAG is often, but not always, unimportant. However, when the non-universal amino acids selenocysteine or pyrrolysine are specified, the selected features are these particular amino acids because of their distinctive properties. (Paradoxically, in a species in which the meaning of UGA, UAA, and UAG has, throughout the body of coding sequences, been reassigned to specify amino acids, their meaning is dynamically redefined, in a context-dependent manner, to specify termination (1, 2).)

Stop codon readthrough is well known in viral decoding, especially of RNA viruses (3). Just as there are select organisms in which RNA editing and ribosomal frameshifting are common, cephalopods (4) and *Euplotes* ciliates (5) respectively, so too is stop codon readthrough unusually common in *Drosophila* (6–8) and related insects (9). However, few instances of stop codon readthrough are known in vertebrate gene decoding. Until relatively recently hardly any instances of experimentally verified conserved mammalian readthrough were known (10, 11), although one of the reported occurrences is at least subject to substantial doubt (12, 13). Recent advances in sequencing technologies paved the way for the advent of ribosome profiling which has identified several potential human readthrough candidates (8, 14, 15). Sequencing advances have also propelled comparative genomics which led to the identification of seven mammalian mRNAs whose expression likely involves stop codon readthrough (7, 16, 17). Subsequent experimental analysis confirmed extended in-frame decoding beyond the annotated stop codon (13, 17–21). Two of these mRNAs, *ACP2* and *SACM1L*, have predicted RNA secondary structures immediately 3′ of their stop codons, and 3′ structural elements are well-known stimulators of functionally utilized stop codon readthrough (22–24). The four mRNAs with the highest readthrough efficiencies, in the tissue culture cells tested so far, are *OPRL1*, *OPRK1*, *AQP4*, and *MAPK10*. Their readthrough efficiencies range from 6 to 17% and all have UGA stop codons immediately followed by CUAG. For these four genes, this motif is conserved not only in mammals but throughout vertebrates, and the importance of the UGA_CUAG motif was confirmed using a systematic mutagenesis approach (17). UGA_CUAG was also subsequently shown to promote readthrough in mRNAs encoding human malate and lactate dehydrogenases (17, 18, 20). Several earlier studies indicated that a cytidine 3′-adjacent to the stop codon influences readthrough in both prokaryotes and eukaryotes (25, 26), but subsequent studies showed that the termination context effect is not limited to a single 3′ nucleotide. The 3′ motif, CARYYA, can stimulate efficient readthrough, especially in plant viruses (27–29). In yeast, a similar sequence (CARNBA) can stimulate readthrough (30). Very recently, using reporters expressed in mammalian cell lines, a comprehensive systematic mutagenesis study identified UGA_CUA among the most highly efficient autonomous readthrough signals (31). Indeed, several alphaviruses employ stop codon readthrough on UGA_CUAG including Middelburg, Ross River, Getah, and also Chikungunya (24). Readthrough has also been identified on UGA_CUA in Mimivirus and Megavirus which are the best characterized representatives of an expanding new family of giant viruses infecting *Acanthamoeba* (32).

A search of all human genes for CUAG immediately following a UGA stop codon indicated that there are 23 instances. Four have positive evolutionary coding potential, as measured by PhyloCSF (33), and these are the four candidates we previously confirmed (17). However, functional readthrough cannot be ruled out for those genes with UGA_CUAG and negative PhyloCSF scores. In fact, readthrough of both malate and lactate dehydrogenases (both harboring UGA_CUAG and both having negative PhyloCSF scores) allows translation of a short peroxisome-targeting motif which has been verified experimentally (18, 20). Here, we investigated stop codon readthrough in all previously untested human mRNAs with UGA_CUAG. Consistent with our previous study showing that UGA-CUA alone can support ∼1.5% readthrough (17), all candidates tested here displayed levels of readthrough ranging from ∼1.3 to 6.7%. The mRNA encoding the vitamin D receptor (VDR)^6^ displayed the highest level of readthrough in this study and was selected for further investigation, however, several other mRNAs, including *ATP10D*, *CDH23*, *DDX58*, *SIRPB1*, and *TMEM86B* also display highly efficient readthrough (∼5.0%).

The VDR is a member of the nuclear receptor superfamily of ligand-inducible transcription factors. Although it is expressed in most tissues, it is most abundant in bone, intestine, kidney, and the parathyroid gland. Consistent with its role as a transcription factor, its expression in tissues and tissue culture cells is low (34). Calcitriol (or 1α,25-dihydroxyvitamin D3) is the ligand for the VDR which mediates the actions of the hormone by ligand-inducible heterodimerization with its partner, retinoid X receptor (RXR). Insufficient concentrations of either calcitriol or the VDR impair calcium and phosphate absorption and hypocalcemia develops which can progress into either rickets in children or else osteomalacia in adults. Dietary vitamin D deficiency is the most common cause of rickets and osteomalacia worldwide. Here we identify a C-terminally extended proteoform of the VDR generated by stop codon readthrough and investigate the effect of this extension on VDR function.

## Results

**Figure 1.**
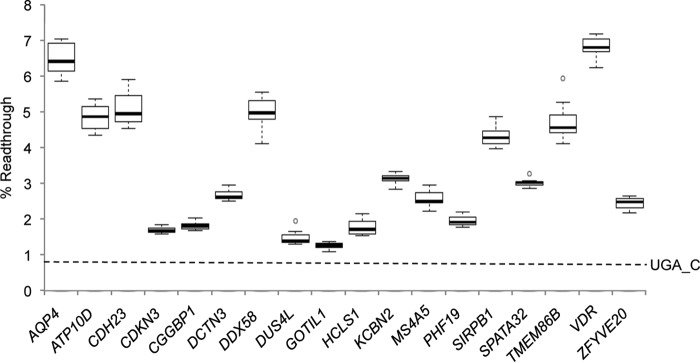
**Readthrough efficiencies of human UGA_CUAG stop codons.** Readthrough efficiencies were determined by dual luciferase assay after transfection of HEK293T cells with dual luciferase reporter constructs consisting of candidate sequences (6 nt 5′ and 12 nt 3′ of stop codon (9 nt 3′ for MS4A5 because of an in-frame stop codon)) shown in Table S1. *AQP4* readthrough has been described previously (17, 21) and is included here as an internal readthrough control. A UGA_C control indicated by a *dashed line* represents background readthrough levels. In each box-whisker plot *center lines* show the medians; *box limits* indicate the 25th and 75th percentiles as determined by R software; *whiskers* extend 1.5 times the interquartile range from the 25th and 75th percentiles; *outliers* are represented by *dots. n* = 12 biological replicates.

Following identification of the UGA_CUAG readthrough motif (17), searches of all human mRNAs for CUAG immediately following a UGA stop codon identified 23 instances (Table S1). This is a significant depletion of this combination of four nucleotides compared with expectations based on the frequencies of the individual nucleotides in those positions immediately following a UGA stop codon (39 expected, one-sided binomial *p* value 0.004). Six of these were previously described and shown by us and others to promote efficient readthrough (13, 17, 18, 20). To experimentally test the remaining 17 potential readthrough candidates, surrounding sequences were cloned in-frame between *Renilla* and firefly luciferase genes. Recently, we described a modification to the classical dual luciferase reporter system (35) that avoids potential distortions, sometimes observed using fused dual reporters, by incorporating “StopGo” sequences on either side of the polylinker (13). The advantage is that reporter activities and/or stabilities are not influenced by the products of the test sequences. HEK293T cells were transfected and lysates assayed by dual luciferase assay. Readthrough efficiencies were determined by comparing relative luciferase activities (firefly/*Renilla*) of test constructs against controls for each construct in which the TGA stop codon is changed to TGG (Trp). All 17 stop codon contexts displayed readthrough efficiencies greater than UGA_C (0.7% readthrough) alone and ranged from 1.3 to 6.7% (Fig. 1).

**Figure 2.**
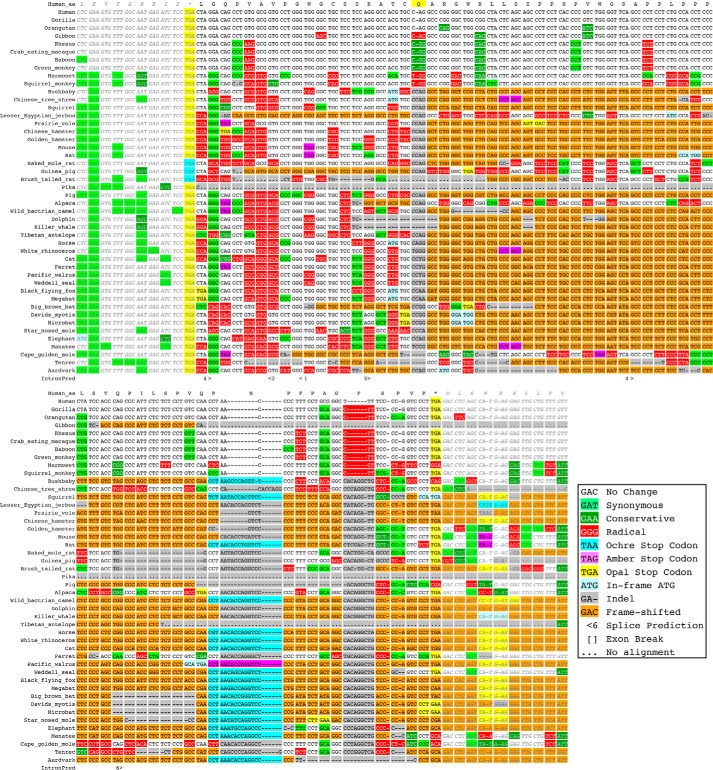
**CodAlignView image of the placental-mammal alignment of the VDR readthrough region with 10-codon context on each side.** A 1-base deletion in the human lineage in the 19th codon (*indicated by highlighted Q*) shifted the theoretical translation frame in Old and New World monkeys from that of *Bushbaby* and more distantly related species. A substitution in the second stop codon in *Marmoset* suggests that readthrough might not have been functional in Old World monkeys until after they split from New World monkeys. The open reading frame is conserved in all of the Old World monkeys except *Gibbon*, in which a 1-base deletion in the 45th codon shifts the reading frame.

Because the *VDR* sequence had the highest readthrough level among these 17 genes, we investigated it further. Bioinformatics analysis provided weak evidence that the 67 amino acid C-terminal readthrough extension to VDR may be functional at the amino acid level in the Old World monkey clade (including apes and human), but probably not in other mammals (Fig. 2). The UGA_CUAG motif is conserved in all the Old and New World monkeys examined, suggesting that translation termination of *VDR* mRNA is not efficient in those species. The second stop codon and reading frame are conserved in gorilla, orangutan, rhesus, crab-eating macaque, baboon, and green monkey. Because the chimp genome assembly has a gap at this locus, we examined the sequence of the *VDR* mRNA and found that the second stop codon and reading frame are conserved in that species as well. In gibbon, there is a 1-base deletion in the 45th codon that disrupts the reading frame. We used PhyloCSF to see if the region between the two stop codons has evolutionary evidence of coding potential in Old World monkeys. The PhyloCSF score of 35.6 for the *VDR* readthrough region alignment in these species is higher than those of same-sized regions 3′ of the stop codon of 97.5% of other transcripts, providing further evidence that translation of the sequence is functional in Old World monkeys. The second stop is not conserved in marmoset, suggesting that the sequence might not be functional in New World monkeys. A 1-base deletion in the 19th codon in the monkey lineage after it split from bushbaby substantially changed the amino acid sequence, and the alignments offer no evidence of functional readthrough in the more distantly related mammals.

Furthermore, inspection of publically available ribosome profiling datasets from experiments in several different human cell lines and tissues compiled in the GWIPs-viz genome browser (36) reveals ribosome density extending 3′ of the annotated *VDR* stop codon which falls off at the next in-frame stop codon, thus providing strong evidence for the existence of *VDR* stop codon readthrough (Fig. S1*A*).

**Figure 3.**
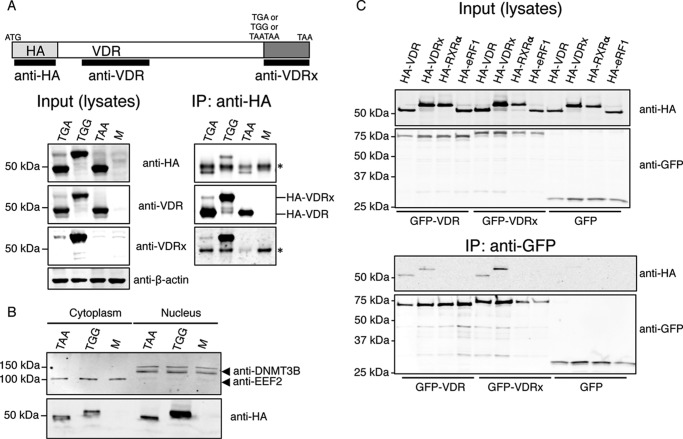
**DRx can translocate to the nucleus and interact with itself and VDR.** *A*, Western blots of protein lysates and anti-HA immunoprecipitates prepared from HEK293T cells either mock-transfected (*M*) or transfected with HA-VDR-TGA (*TGA*), HA-VDR-TGG (*TGG*), or HA-VDR-TAATAA (*TAA*) as indicated. Anti-VDRx is a custom polyclonal antibody raised in rabbit against the full 67 amino acid VDR readthrough peptide. The *asterisk* indicates immunodetection of the IgG heavy chain. *B*, Western blots of cytoplasmic and nuclear fractions from HEK293T cells either mock-transfected (*M*) or transfected with HA-VDR-TAATAA (*TAA*) or HA-VDR-TGG (*TGG*) as indicated. *EEF2* is eukaryotic elongation factor 2 located predominantly in the cytoplasm and *DNMT3B* is DNA methyltransferase 3 beta located predominantly in the nucleus. *C*, Western blots of protein lysates and anti-GFP immunoprecipitates prepared from HEK293T cells co-transfected with the indicated expression constructs. *HA-eRF1* is an HA-tagged eukaryotic release factor 1 used here as a negative control.

The readthrough assays in Fig. 1 suggest that ∼7% of the ribosomes translating the *VDR* mRNA decode its UGA stop codon as a sense codon, thus extending VDR at its C terminus by an additional 67 amino acids to generate VDRx (extended: Fig. S1*B*). To test *VDR* readthrough in the context of the full coding sequence we transfected HEK293T cells with constructs encoding an N-terminal HA-tagged wildtype VDR that also included ∼200 nt of 3′UTR (HA-VDR-TGA). Control constructs in which the TGA stop codon was changed to either a sense codon (HA-VDR-TGG: readthrough positive control) or to a nonreadthrough double stop codon (HA-VDR-TAATAA: readthrough negative control), were also transfected. Anti-HA immunoprecipitates were immunoblotted with a commercially available anti-VDR and a custom antibody raised against the 67 amino acid VDR readthrough peptide (anti-VDRx). In cells transfected with HA-VDR-TGA and HA-VDR-TAATAA, a protein of ∼50 kDa, corresponding to HA-tagged canonical VDR, was detected by anti-VDR but not by anti-VDRx (Fig. 3*A*). Both anti-VDR and anti-VDRx also detected a less-abundant protein of ∼55 kDa in cells expressing HA-VDR-TGA and this protein co-migrates with the major protein detected in cells expressing HA-VDR-TGG (Fig. 3*A*). Similar results were also observed for VDR constructs tagged with GFP instead of HA (Fig. S2). Together these immunoprecipitation experiments provide further evidence for the utilization of stop codon readthrough during *VDR* decoding.

The extensions of N-terminal or C-terminal extended proteins sometimes target proteins to subcellular compartments (18, 20, 37, 38). Subcellular targeting prediction software did not reveal known signals within the VDR extension. In addition, live cell imaging of HeLa cells expressing GFP with the 67 amino acid VDR extension fused to its C terminus displayed a subcellular distribution similar to GFP alone (Fig. S3). Normally, hormone receptors like the VDR reside in both the cytoplasm and the nucleus (39). To determine whether VDRx is located in the cytoplasm and nucleus we transfected HeLa cells with constructs expressing GFP N-terminal fusions of VDRx (TGA to TGG) and a mutant VDR (TGA to TAATAA) where readthrough is undetectable (Fig. S2). Live cell imaging in either resting cells or cells stimulated with calcitriol for 10 min revealed that the subcellular distribution of VDR and VDRx are almost identical (Fig. S3), with both displaying cytoplasmic and nuclear localization when resting and when stimulated. Furthermore, anti-HA immunoblots of nuclear and cytoplasmic fractions isolated from cells transfected with HA-VDR or HA-VDRx provide further support that, like VDR, VDRx can localize in both the cytoplasm and nucleus (Fig. 3*B*). Together, these data suggest that the VDR extension does not dramatically alter or target VDRx to a discernible subcellular location.

Next we explored the possibility that VDRx can form homodimers and heterodimers with VDR and RXRα by co-immunoprecipitation experiments from cells co-transfected with epitope-tagged variants of VDRx, VDR, and RXRα. GFP-VDRx co-immunoprecipitates with both HA-VDR and HA-VDRx (Fig. 3*C*). In addition, GFP-VDR also co-immunoprecipitates with both HA-VDR and HA-VDRx. We could not co-immunoprecipitate HA-RXRα with either GFP-VDR or GFP-VDRx; presumably, the gentle lysis required for co-immunoprecipitation experiments limits extraction from the nucleus where VDR-RXRα heterodimers are predominantly localized.

**Figure 4.**
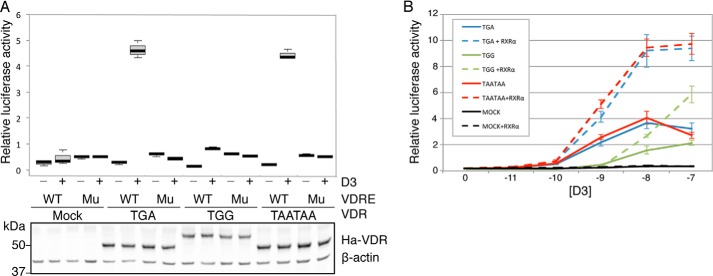
**VDRx is less responsive than VDR to calcitriol.** *A*, relative luciferase activities determined by dual luciferase assay after co-transfection of HEK293T cells with plasmids expressing HA-tagged VDR proteins (or mock transfected) as indicated, together with firefly luciferase reporter constructs driven by a minimal promoter with either tandem VDR elements (VDRE) from the rat osteocalcin gene (*WT*) or control firefly luciferase reporters harboring mutated VDREs (*Mu*). A *Renilla* luciferase plasmid was also co-transfected to allow firefly luciferase activity normalization. Transfectants that were treated with vehicle (ethanol) control are indicated by the *minus*, and the *plus* indicates activities from calcitriol-stimulated (1 nm) cells. Representative anti-HA Western blot of the same lysates are shown under the histogram. In each box-whisker plot *center lines* show the medians; *box limits* indicate the 25th and 75th percentiles as determined by R software; *whiskers* extend 1.5 times the interquartile range from the 25th and 75th percentiles; and *outliers* are represented by dots. *n* = 4 biological replicates. *B*, relative luciferase activities determined by dual luciferase assay of HEK293T cells transfected as in (*A*) then treated with varying concentrations of calcitriol as indicated for 24 h. *Dashed lines* indicate relative luciferase activities when HA-RXRα was co-transfected along with VDR variants. *Error bars*, mean ± S.D. are shown from *n* = 8 biological replicates.

The ligand-binding domain of VDR is encoded by amino acids at its extreme C terminus (40). Because VDRx extends the VDR C terminus by an additional 67 amino acids, we set out to determine whether VDRx responds to calcitriol similarly to VDR. Firefly luciferase reporter constructs driven by a minimal promoter with tandem VDR elements (VDRE) from the rat osteocalcin gene were co-transfected with each of the HA-tagged VDR constructs described above and then stimulated with 1 nm calcitriol. Control firefly luciferase reporter constructs harboring mutated VDREs were also included. Calcitriol stimulated relative luciferase activities 5- to 7-fold in cells co-transfected with either HA-VDR-TGA or HA-VDR-TAATAA, whereas cells co-transfected with HA-VDR-TGG (VDRx) did not respond to calcitriol (Fig. 4*A*, *upper panel*). This clear inability of VDRx to transactivate in response to calcitriol cannot be accounted for solely by its slightly lower steady state levels (Fig. 4*A*, *lower panel*). Whether VDRx is completely unresponsive to calcitriol or just less responsive was examined by transactivation experiments using a range of calcitriol concentrations. Here, almost 100 times more calcitriol is required for HA-VDR-TGG to elicit the same response as either HA-VDR-TGA or HA-VDR-TAATAA, indicating that the ability of VDRx to transactivate is much less responsive to calcitriol than for VDR (*solid lines* in Fig. 4*B*). However, transactivation by VDRx is still much higher than in mock-transfected (empty vector) cells where only endogenous levels of VDR are expressed, indicating that VDRx retains some capacity to bind calcitriol and must also retain DNA-binding capability.

Although we could not detect VDR or VDRx complexed with RXRα by co-immunoprecipitation (Fig. 3*C*), co-expressing HA-RXRα together with VDR variants resulted in dramatic calcitriol responsive increases in VDRE-reporter transactivation regardless of which VDR variant is overexpressed (*dashed lines* in Fig. 4*B*). Although transactivation by VDRx plus RXRα is still reduced compared with VDR with RXRα, it is clear that the VDR C-terminal extension does not completely abrogate heterodimerization with RXRα.

## Discussion

In eukaryotes several factors are known to dramatically affect translation termination efficiency and therefore stop codon readthrough. These include the nucleotide sequences surrounding the stop codon (25, 41–46), transacting factors (47–49), abundance of near-cognate tRNAs (50–52), abundance and/or modifications of release factors (53–57), and the presence of mRNA secondary structures (22–24, 58–60). In addition, a role for eIF5A in eukaryotic translation termination has been recently reported (61, 62). Interestingly, the amino acid specified is influenced not only by the identity of the stop codon but also local context and tRNA availability, which are likely important considerations for potential treatments of disease-causing premature termination codons (51, 52).

Following on from our previous studies which identified a novel stop codon readthrough context in higher eukaryotes (13, 17), we show here that all human genes with this stop codon motif display readthrough. However, although the UGA_CUAG motif alone seems to be sufficient for ∼1.3% readthrough, additional local sequences must also be important because the readthrough efficiencies of mRNAs containing this motif range from ∼1.3% (*GOTIL1* this study) up to ∼17% *OPRL1* (13). Given that we tested only 21 nt surrounding the stop codon, the >10-fold difference between the lowest and highest readthrough contexts must reside in this small region. We are currently attempting to identify this additional readthrough stimulator.

How stop codon context and/or nearby RNA secondary structures influence the competition between productive near-cognate tRNA and eukaryotic factor 1 (eRF1) recognition of stop codons is still unknown. Recent cryo-EM structures of eukaryotic ribosomes complexed with eRF1 docked on a stop codon revealed that nucleotide A1825 of 18S rRNA is flipped so that it stacks on the second and third stop codon bases. This formation pulls the 3′ base adjacent to the stop codon into the A-site forming a 4-base U-turn where it is stabilized by stacking against G626 of 18S rRNA (63, 64). Stacking with G626 would be more stable for purines, which may explain their statistical bias at the +4 position (25), but still does not explain why cytidine at this position appears to reduce termination efficiency more than uracil. One possible explanation for how stop codon contexts influence termination could be that mRNA sequences surrounding the stop codon and/or an RNA secondary structure may restrict the formation of the U-turn within the A-site that appears to be necessary for stop codon recognition by eRF1. Perhaps mRNA bases 3′ of the stop codon pair with rRNA bases within the mRNA entrance tunnel, as has been considered for some cases of alphavirus-programmed ribosomal frameshifting (65).

Of the 17 readthrough candidates tested in this study we observed highest readthrough efficiencies for VDR (∼7%) using a novel dual luciferase reporter system (Fig. 1). We also confirm VDR readthrough by Western blotting with commercially available VDR antibodies as well as a custom antibody raised against the 67 amino acid VDR extension (Fig. 3). Evidence for endogenous VDR readthrough is provided by the analysis of ribosome profiles, indicating that ribosome protected fragments map to the VDR transcript immediately 3′ of the annotated stop codon but not beyond the next in-frame stop codon (Fig. S1). Overall we provide strong evidence that some ribosomes read through the stop codon of the VDR coding sequence to generate a C-terminally extended proteoform, VDRx.

Several studies have shown that readthrough can generate dual-targeted proteoforms (18, 20, 37) but here, TargetP analysis of VDR did not reveal subcellular targeting motifs within the readthrough extension. Consistent with this prediction, live cell imaging of fluorescently labeled VDRx indicates that its subcellular localization is identical to VDR (Fig. S3), thus suggesting that there are no cryptic targeting motifs within the new VDRx C terminus. We also used the Predictor of Natural Disordered Regions (66) algorithm to infer ordered and disordered segments in the VDRx extension which were inferred to be largely disordered. No significant homology was found between the VDRx readthrough peptide sequences and structural domains within the InterProScan (67) or NCBI conserved domains databases (68).

Other protein isoforms of the human VDR have been identified previously by others. A common polymorphism that changes the annotated start codon produces a VDR isoform initiated from a 3′ AUG codon with a 3–amino acid N-terminal truncation (69) which has been associated with elevated transactivation activity (70, 71). Another VDR isoform with a 50–amino acid N-terminal extension termed VDRB1 is generated by alternative splicing (72, 73). Because the predicted molecular weight of VDRB1 is almost identical to that predicted for VDRx, caution should be exercised when interpreting immunoblots that use antibodies directed against the VDR coding sequence. VDRB1 only differs from VDR by its extended N terminus, which suggests the likely existence of a C-terminal extended proteoform of VDRB1 (VDRB1x) generated by stop codon readthrough.

The function of VDRx is still unclear and requires further investigation. Phylogenetic approaches allow identification of evolutionarily conserved functional readthrough but cannot reveal instances that either emerged recently or are not under strong evolutionary selection. Although bioinformatic analysis argues against strong evolutionary selection of the VDR extension beyond Old World monkeys, our studies also indicate that the VDR extension does appear to have an overall negative effect on VDR function because VDRx is less able to elicit a transcriptional response to calcitriol. There are several possibilities for why VDRx elicits a reduced response to calcitriol. Given the proximity of the VDR extension to the ligand-binding domain it would not be surprising if this juxtaposition results in impaired binding to calcitriol. However, other possibilities include either reduced ability to heterodimerize with RXRα or insufficient recognition of VDREs within the promoters of its transcriptional targets. It should be noted that our findings (Fig. 3*C*) suggest that VDRx can form heterodimers with VDR, which could potentially antagonize VDR action.

It seems likely that the VDR readthrough extension has recently emerged and can improve organism fitness under some, as yet unidentified, physiological condition that appears to be specific for humans and Old World monkeys. Perhaps another ligand for VDR exists in higher primates that has higher affinity to VDRx than VDR. Interestingly, there is some precedent for other recently emerged VDR variations that include three primate-specific exons (5′ leader) (74).

## Experimental procedures

### Plasmids

For the generation of dual luciferase expression constructs, overlapping oligonucleotide pairs (Integrated DNA Technologies (IDT)) containing sequence flanking the stop codons (6 nt 5′ and 12 nt 3′) (see Table S2) of the predicted readthrough candidates were annealed and ligated with PspXI/BglII-digested pSGDluc (13).

The HA-VDR-TGA expression clone was made by PCR amplifying the VDR coding sequence plus 200 nucleotides of 3′UTR from HEK293T cDNA and then cloned BamHI/XbaI in-frame with the influenza HA tag in pcDNA3-HA (Invitrogen). HA-VDR-TGG and HA-VDR-TAATAA were generated by two-step PCR mutagenesis using HA-VDR-TGA as template (see Table S2 for PCR primers). RXRα was synthesized by IDT as a G Block and digested with incorporated 5′ BglII and 3′ XbaI restriction sites then ligated with BamHI/XbaI-digested pcDNA3-HA to generate HA-RXRα. GFP-VDR fusion constructs were made by subcloning the VDR-TGA, VDR-TGG, and VDR-TAATAA cassettes from the pcDNA3-HA constructs just described into pEGFP-C3 (Clontech).

Wildtype (WT) and mutant VDRE–firefly luciferase fusions were generated by restricting WT and mutant G Blocks (IDT) (see Table S2 for sequences) with SacI/BglII then ligating with SacI/BglII-restricted pDLuc (75). SacI/BglII digestion of pDLuc removes the SV40 promoter and *Renilla* luciferase. All constructs were verified by DNA sequencing.

### Cell culture and transfections

HEK293T cells (ATCC) and HeLa cells (ATCC) were maintained in DMEM supplemented with 10% FBS, 1 mm l-glutamine, and antibiotics. HEK293T cells were transfected with Lipofectamine 2000 reagent (Invitrogen), using the 1-day protocol in which suspended cells are added directly to the DNA complexes in half-area 96-well plates. For transfections shown in Fig. 1 the following were added to each well: 25 ng of each plasmid plus 0.2 μl Lipofectamine 2000 in 25 μl Opti-Mem (Gibco Laboratories). The transfecting DNA complexes in each well were incubated with 4 × 10^4^ cells suspended in 50 μl DMEM plus 10% FBS at 37 °C in 5% CO_2_ for 24 h.

For transactivation experiments shown in Fig. 4*A* the following amounts of DNA were added to each well: 20 ng of VDRE-firefly, 5 ng *Renilla* expressing plasmid, and 20 ng of either HA-tagged VDR expressing plasmid or empty vector (MOCK). For transactivation experiments shown in Fig. 4*B* the following amounts of DNA were added to each well: 20 ng of VDRE-firefly, 5 ng *Renilla* expressing plasmid, and 10 ng of each HA-tagged VDR plus 10 ng of either HA-RXRα or empty vector. The transfecting DNA complexes in each well were incubated with 4 × 10^4^ cells suspended in 25 μl DMEM plus 10% FBS at 37 °C in 5% CO_2_ for 1 h before the addition of DMEM plus calcitriol (Enzo Life Sciences) or ethanol control at the indicated final concentrations for a further 24 h.

### Dual luciferase assay

Firefly and *Renilla* luciferase activities were determined using the Dual Luciferase Stop & Glo® Reporter Assay System (Promega). Relative light units were measured on a Veritas Microplate Luminometer with two injectors (Turner Biosystems). Transfected cells were lysed in 12.6 μl of 1× passive lysis buffer (PLB) and light emission was measured following injection of 25 μl of either *Renilla* or firefly luciferase substrate. Readthrough efficiencies (% readthrough) were determined by calculating relative luciferase activities (firefly/*Renilla*) of TGA constructs and dividing by relative luciferase activities from replicate wells of control TGG constructs. The number of biological replicates for each experiment is indicated in each figure legend. Where possible all data points are presented, otherwise mean ± S.D. is presented.

### Western blot analysis

Cells were transfected in 6-well plates using Lipofectamine 2000 reagent, again using the 1-day protocol described above, with 1 μg of each indicated plasmid. The transfecting DNA complexes in each well were incubated with 10^6^ HEK293T cells suspended in 3 ml DMEM plus 10% FBS and incubated overnight at 37 °C in 5% CO_2_. Transfected cells were lysed in 100 μl 1× PLB. Cytoplasmic and nuclear fractions were isolated using the REAP protocol (76). Proteins were resolved by SDS-PAGE and transferred to nitrocellulose membranes (Protran), which were incubated at 4 °C overnight with primary antibodies. Immunoreactive bands were detected on membranes after incubation with appropriate fluorescently labeled secondary antibodies using a LI-COR Odyssey® Infrared Imaging Scanner.

### Immunoprecipitation

Cells were lysed in 700 μl PLB and then incubated with 20 μl of protein G agarose beads plus anti-HA (3 μg) overnight at 4 °C with gentle rocking. The beads were washed with ice-cold 1× PLB and then removed from the beads by boiling for 5 min in 2× SDS-PAGE sample buffer for SDS-PAGE and Western blotting. For GFP immunoprecipitation, GFP-Trap (Chromtek) was used following the manufacturer's instructions. Briefly, cells were lysed in 100 μl of Nonidet P-40 lysis buffer (10 mm Tris-Cl, pH 7.5, 150 mm NaCl, 0.5 mm EDTA, 0.5% Nonidet P-40) then 95 μl of lysate was diluted to 700 μl in dilution buffer (10 mm Tris-Cl, pH 7.5, 150 mm NaCl, 0.5 mm EDTA) before incubation with 20 μl of GFP-Trap beads for 1 h at 4 °C with gentle rocking. The beads were washed with ice-cold dilution buffer and then removed from the beads by boiling for 5 min in 2× SDS-PAGE sample buffer for SDS-PAGE and Western blotting.

### Fluorescence microscopy

Live cell imaging was performed as described before (77) using an inverted Axiovert 200 fluorescence microscope (Zeiss), equipped with 100×/1.4 Plan-Apochromat oil-immersion objective (Zeiss), pulsed excitation module (470 nm, 390 nm LEDs), bandpass filters 510–560 nm (EGFP) and 417–477 nm (Hoechst 33342) and gated CCD camera (LaVision BioTec). Briefly, HeLa cells were seeded onto 8-well chambers precoated with a mixture of collagen IV and poly-d-lysine (Ibidi), allowed to attach (24 h) and forward transfected for 24 h with plasmids encoding GFP-VDR fusions as indicated. Prior to live imaging, cells were counterstained with Hoecsht 33342 (1 μm, 30 min). Fluorescence images were collected before (resting) and after stimulation with calcitriol (10^−7^ m, 10 min, 37 °C). Images were exported using ImSpector software (LaVision BioTec) and combined in Adobe Illustrator CS2.

### Antibodies

An affinity-purified rabbit polyclonal antibody to the 67 amino acid VDR readthrough peptide was prepared by Proteintech to generate anti-VDRx. The following commercially available antibodies were also used. Rabbit anti-VDR (Abcam, ab109234), mouse anti-HA (Covance, clone 16B12), mouse anti-β-actin (Sigma, A3853), rabbit anti-DNMT3B (Abcam, ab79822), and rabbit anti-EEF2 (Cell Signaling Technology, 2332).

### PhyloCSF and bioinformatics analysis

Human transcript models were obtained from GENCODE version 16 (78). All protein-coding transcripts were searched for the UGA_CUAG readthrough motif at the end of the annotated coding sequence. PhyloCSF was run using the 29mammals parameters on the region between the annotated stop codon and the next in-frame stop codon, not including either stop codon, using the 29 placental mammals subset of the 46-vertebrate hg19 whole-genome MULTIZ alignments (79), which were obtained from the UCSC genome browser (80). To compute the evolutionary coding potential of the VDR readthrough region in Old World monkeys, we computed PhyloCSF using the 58 mammals parameters on the subset of the 100-vertebrates hg19 alignments consisting of the species human, gorilla, orangutan, gibbon, rhesus, crab-eating macaque, baboon, and green monkey. To estimate the significance of this score, we similarly computed PhyloCSF scores of the 66 codons 3′ of each unique annotated stop codon. For the sequence of the Chimp VDR mRNA we used NCBI Reference Sequence XM_016923548.1 obtained from https://www.ncbi.nlm.nih.gov/nucleotide/1034096372. Alignments were examined using CodAlignView (I. Jungreis, M. Lin, C. Chan, and M. Kellis, CodAlignView: The Codon Alignment Viewer, available from http://data.broadinstitute.org/compbio1/cav.php).^7^

## Author contributions

G. L. and J. F. A. conceived and coordinated the study and wrote the paper. G. L. designed, performed, and analyzed the experiments shown in Figures 1, 3, and 4 and Figures S1 and S2. I. J., I. P. I., and M. K. performed motif searches and bioinformatic analysis shown in Figure 2. I. T. generated the HA-VDR constructs and M. P. provided technical assistance. R. I. D. performed and analyzed the experiments shown in Figure S3. All authors reviewed the results and approved the final version of the manuscript.

## Supplementary Material

Supporting Information

## Footnotes

7 Please note that the JBC is not responsible for the long-term archiving and maintenance of this site or any other third party hosted site.

## Footnotes

6 The abbreviations used are:

VDR: vitamin D receptor
RXR: retinoid X receptor
nt: nucleotide
VDRE: VDR elements
PLB: passive lysis buffer.
