# Supplementary material for: Stop codon readthrough generates a C-terminally extended variant of the human vitamin D receptor with reduced calcitriol response
Source: J Biol Chem. 2018 Jan 31;293(12):4434–44. doi: 10.1074/jbc.M117.818526 (PMC5868278; doi:10.1074/jbc.M117.818526)
Supplement: Supporting Information [file supp_293_12_4434_v2_index.html]

Stop codon readthrough generates a C-terminally extended variant of the human vitamin D receptor with reduced calcitriol response — Novel variant of the human vitamin D receptor — Supporting Information 

# Stop codon readthrough generates a C-terminally extended variant of the human vitamin D receptor with reduced calcitriol response

## Supporting Information

- Supplemental Tables and Figures
