# Supplementary material for: Stop codon readthrough generates a C-terminally extended variant of the human vitamin D receptor with reduced calcitriol response
Source: J Biol Chem. 2018 Jan 31;293(12):4434–44. doi: 10.1074/jbc.M117.818526 (PMC5868278; doi:10.1074/jbc.M117.818526)

Stop codon readthrough generates a C-terminally extended variant of the human vitamin D receptor with reduced calcitriol response

Gary Loughran[1,*], Irwin Jungreis[2], Ioanna Tzani[1,3], Michael Power[1,4], Ruslan I. Dmitriev[1,] Ivaylo P Ivanov[1,5], Manolis Kellis[2], John F Atkins[1,6,*].

Table S1
Table S2
Figure S1
Figure S2
Figure S3

Table S1. Details of all human mRNAs with coding sequences ending in UGA_CUAG indicating the number of predicted readthrough codons as well as the sequences tested in this study.

GeneId	GeneName	Codons	CodAlignView	Sequence tested this study	Amino acids
ENSG00000171885.9	*AQP4*	29	CodAlignView	TCA GTA TGA CTA GAA GAT CGC	SV-LEDR
ENSG00000145246.9	*ATP10D*	16	CodAlignView	AAT GCA TGA CTA GAG TCT TGC	NA-LESC
ENSG00000107736.15	*CDH23*	9	CodAlignView	GAG CTG TGA CTA GAC AGG GAA	EL-LDRE
ENSG00000100526.14	*CDKN3*	20	CodAlignView	CTA CGG TGA CTA GTT TAT TTT	LR-LVYF
ENSG00000163320.6	*CGGBP1*	31	CodAlignView	GAT TGT TGA CTA GGA GGT TAC	DC-LGGY
ENSG00000137100.11	*DCTN3*	51	CodAlignView	GGG AGA TGA CTA GAA GCG AAG	GR-LEAK
ENSG00000107201.5	*DDX58*	6	CodAlignView	TCC TTC TGA CTA GTA ATG CTG	SF-LVML
ENSG00000105865.6	*DUS4L*	12	CodAlignView	GGC ATT TGA CTA GAC TTC CCA	GI-LDFP
ENSG00000169154.4	*GOT1L1*	56	CodAlignView	CTT CAC TGA CTA GGT GAC CAG	LH-LGDQ
ENSG00000180353.6	*HCLS1*	30	CodAlignView	CTG GAG TGA CTA GAG CTC ACT	LE-LELT
ENSG00000182674.5	*KCNB2*	8	CodAlignView	AGC ATG TGA CTA GTT ACA AAA	SM-LVTK
ENSG00000166930.2	*MS4A5*	4	CodAlignView	TGT TGT TGA CTA GCA CTG TGG*	CC-LAL-
ENSG00000119403.9	*PHF19*	157	CodAlignView	CCT TAC TGA CTA GCC CCC GGG	PY-LAPG
ENSG00000101307.11	*SIRPB1*	91	CodAlignView	AAG GCC TGA CTA GTC CTT GAT	KA-LVLD
ENSG00000184361.8	*SPATA32*	105	CodAlignView	GAG AAA TGA CTA GAC AGA ACC	EK-LDRT
ENSG00000180089.4	*TMEM86B*	110	CodAlignView	ACT GAC TGA CTA GGG AGC TTG	TD-LGSL
ENSG00000111424.6	*VDR*	67	CodAlignView	ATC TCC TGA CTA GGA CAG CCT	IS-LGQP
ENSG00000131381.8	*ZFYVE20*	39	CodAlignView	AAC AGA TGA CTA GTG TTT GTC	NR-LVFV
	UGA_C			T GCA TGA CAT CTT A	

Table S2. Sequences of oligonucleotides and G Blocks used in this study.

NAME	SEQUENCE (5'-3')
AQP4 S	TCGAGATCAGTATGACTAGAAGATCGCA
AQP4 AS	GATCTGCGATCTTCTAGTCATACTGATC
AQP4 UGG S	TCGAGATCAGTATGGCTAGAAGATCGCA
AQP4 UGG AS	GATCTGCGATCTTCTAGCCATACTGATC
ATP10D S	TCGAGAAATGCATGACTAGAGTCTTGCA
ATP10D AS	GATCTGCAAGACTCTAGTCATGCATTTC
ATP10D UGG S	TCGAGAAATGCATGGCTAGAGTCTTGCA
ATP10D UGG AS	GATCTGCAAGACTCTAGCCATGCATTTC
CDH23 S	TCGAGAGAGCTGTGACTAGACAGGGAAA
CDH23 AS	GATCTTTCCCTGTCTAGTCACAGCTCTC
CDH23 UGG S	TCGAGAGAGCTGTGGCTAGACAGGGAAA
CDH23 UGG AS	GATCTTTCCCTGTCTAGCCACAGCTCTC
CDKN3 S	TCGAGACTACGGTGACTAGTTTATTTTA
CDKN3 AS	GATCTAAAATAAACTAGTCACCGTAGTC
CDKN3 UGG S	TCGAGACTACGGTGGCTAGTTTATTTTA
CDKN3 UGG AS	GATCTAAAATAAACTAGCCACCGTAGTC
CGGBP1 S	TCGAGAGATTGTTGACTAGGAGGTTACA
CGGBP1 AS	GATCTGTAACCTCCTAGTCAACAATCTC
CGGBP1 UGG S	TCGAGAGATTGTTGGCTAGGAGGTTACA
CGGBP1 UGG AS	GATCTGTAACCTCCTAGCCAACAATCTC
DCTN3 S	TCGAGAGGGAGATGACTAGAAGCGAAGA
DCTN3 AS	GATCTCTTCGCTTCTAGTCATCTCCCTC
DCTN3 UGG S	TCGAGAGGGAGATGGCTAGAAGCGAAGA
DCTN3 UGG AS	GATCTCTTCGCTTCTAGCCATCTCCCTC
DDX58 S	TCGAGATCCTTCTGACTAGTAATGCTGA
DDX58 AS	GATCTCAGCATTACTAGTCAGAAGGATC
DDX58 UGG S	TCGAGATCCTTCTGGCTAGTAATGCTGA
DDX58 UGG AS	GATCTCAGCATTACTAGCCAGAAGGATC
DUS4L S	TCGAGAGGCATTTGACTAGACTTCCCAA
DUS4L AS	GATCTTGGGAAGTCTAGTCAAATGCCTC
DUS4L UGG S	TCGAGAGGCATTTGGCTAGACTTCCCAA
DUS4L UGG AS	GATCTTGGGAAGTCTAGCCAAATGCCTC
GOT1L1 S	TCGAGACTTCACTGACTAGGTGACCAGA
GOT1L1 AS	GATCTCTGGTCACCTAGTCAGTGAAGTC
GOT1L1 UGG S	TCGAGACTTCACTGGCTAGGTGACCAGA
GOT1L1 UGG AS	GATCTCTGGTCACCTAGCCAGTGAAGTC
HCLS1 S	TCGAGACTGGAGTGACTAGAGCTCACTA
HCLS1 AS	GATCTAGTGAGCTCTAGTCACTCCAGTC
HCLS1 UGG S	TCGAGACTGGAGTGGCTAGAGCTCACTA
HCLS1 UGG AS	GATCTAGTGAGCTCTAGCCACTCCAGTC
KCNB2 S	TCGAGAAGCATGTGACTAGTTACAAAAA
KCNB2 AS	GATCTTTTTGTAACTAGTCACATGCTTC
KCNB2 UGG S	TCGAGAAGCATGTGGCTAGTTACAAAAA
KCNB2 UGG AS	GATCTTTTTGTAACTAGCCACATGCTTC
MS4A5 S	TCGAGATGTTGTTGACTAGCACTGA
MS4A5 AS	GATCTCAGTGCTAGTCAACAACATC
MS4A5 S UGG	TCGAGATGTTGTTGGCTAGCACTGA

MS4A5 AS UGG	GATCTCAGTGCTAGCCAACAACATC
PHF19 S	TCGAGACCTTACTGACTAGCCCCCGGGA
PHF19 AS	GATCTCCCGGGGGCTAGTCAGTAAGGTC
PHF19 UGG S	TCGAGACCTTACTGGCTAGCCCCCGGGA
PHF19 UGG AS	GATCTCCCGGGGGCTAGCCAGTAAGGTC
SIRPB1 S	TCGAGAAAGGCCTGACTAGTCCTTGATA
SIRPB1 AS	GATCTATCAAGGACTAGTCAGGCCTTTC
SIRPB1 UGG S	TCGAGAAAGGCCTGGCTAGTCCTTGATA
SIRPB1 UGG AS	GATCTATCAAGGACTAGCCAGGCCTTTC
SPATA32 S	TCGAGAGAGAAATGACTAGACAGAACCA
SPATA32 AS	GATCTGGTTCTGTCTAGTCATTTCTCTC
SPATA32 UGG S	TCGAGAGAGAAATGGCTAGACAGAACCA
SPATA32 UGG AS	GATCTGGTTCTGTCTAGCCATTTCTCTC
TMEM86B S	TCGAGAACTGACTGACTAGGGAGCTTGA
TMEM86B AS	GATCTCAAGCTCCCTAGTCAGTCAGTTC
TMEM86B UGG S	TCGAGAACTGACTGGCTAGGGAGCTTGA
TMEM86B UGG AS	GATCTCAAGCTCCCTAGCCAGTCAGTTC
VDR S	TCGAGAATCTCCTGACTAGGACAGCCTA
VDR AS	GATCTAGGCTGTCCTAGTCAGGAGATTC
VDR UGG S	TCGAGAATCTCCTGGCTAGGACAGCCTA
VDR UGG AS	GATCTAGGCTGTCCTAGCCAGGAGATTC
ZFYVE20 S	TCGAGAAACAGATGACTAGTGTTTGTCA
ZFYVE20 AS	GATCTGACAAACACTAGTCATCTGTTTC
ZFYVE20 UGG S	TCGAGAAACAGATGGCTAGTGTTTGTCA
ZFYVE20 UGG AS	GATCTGACAAACACTAGCCATCTGTTTC
UGA_C neg co S	TCGAGTGCATGACATCTTA
UGA_C neg co AS	GATCTAAGATGTCATGCAC
UGG_C neg co S	TCGAGTGCATGGGATCTTA
UGG_C neg co AS	GATCTAAGATCCCATGCAC
HA-VDR BamH1 S	ATAAGGATCCTGAGGCAATGGCGGCCAGCACTTCC
HA-VDR XbaI AS	TTATTCTAGATTATCAAGGGACCGGGGAAAAGCCCGC
HA-VDR UGA-UGG S	GGCAATGAGATCTCCTGGCTAGGACAGCCTGTG
HA-VDR UGA-UGG AS	CACAGGCTGTCCTAGCCAGGAGATCTCATTGCC
HA-VDR UGA_CUA-UAA_UAA S	GGCAATGAGATCTCCTAATAAGGACAGCCTGTG
HA-VDR UGA_CUA-UAA_UAA AS	CACAGGCTGTCCTTATTAGGAGATCTCATT
VDRE WT G Block	ATAAGAGCTCGCTAGCTTCGAGGATATCAGATCAGCGCAGCACCATGGCCTGAAA
	TAACCTCTGAAAGAGGAACTTGGTTAGGTACCTTCTGAGGCGGAAAGAACCAGCT
	GTGGAATGTGTGTCAGTTAGGGTGTGGAAAGTCCCCAGGCTCCCCAGCAGGCAGA
	AGTATGCAAAGCATGCATCTCAATTAGTCAGCAACCAGGTAAGCTTCACTGGGTG
	AATGAGGACATTACCACTGGGTGAATGAGGACATTACGAATTCTCGAGGTCGACA
	GCGGAGACTCTAGAGGGTATATAGCTTGGCAATCCGGTACTGTTGGTAAGCCACC
	ATGAGATCTATAA
VDRE Mu G Block	ATAAGAGCTCGCTAGCTTCGAGGATATCAGATCAGCGCAGCACCATGGCCTGAAA
	TAACCTCTGAAAGAGGAACTTGGTTAGGTACCTTCTGAGGCGGAAAGAACCAGCT
	GTGGAATGTGTGTCAGTTAGGGTGTGGAAAGTCCCCAGGCTCCCCAGCAGGCAGA
	AGTATGCAAAGCATGCATCTCAATTAGTCAGCAACCAGGTAAGCTTCACTCCCAC
	TATGTCCTGATTACCACTCCCACTATGTCCTGATTACGAATTCTCGAGGTCGACA
	GCGGAGACTCTAGAGGGTATATAGCTTGGCAATCCGGTACTGTTGGTAAGCCACC
	ATGAGATCTATAA

Figure S1. (A) Screenshot from GWIP-viz (hg38) of sequences surrounding the annotated *VDR* stop codon. RNAseq alignments are shown as green histograms and the positions of ribosome A-sites inferred from alignments of ribosome-protected fragments (Riboseq) are shown as red columns. The data are aggregated from 20 ribosome profiling studies carried out in several human cultured cells. The number of reads at each nucleotide position is indicated on the y-axis. (B) Amino acid sequence of human VDRx with the readthrough extension in red font.

A

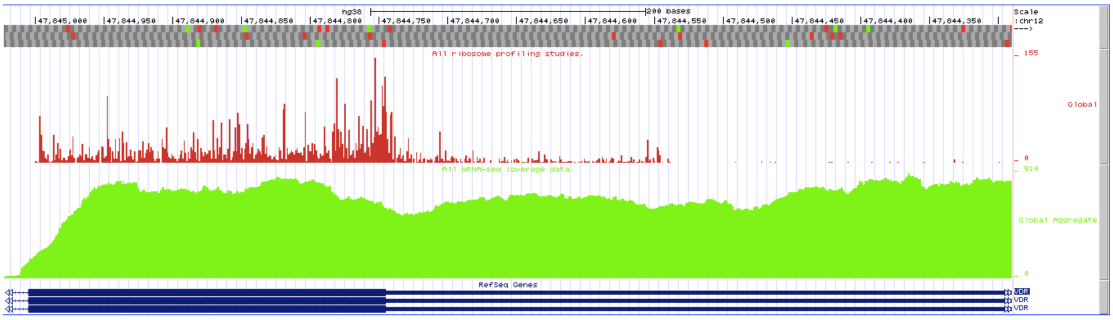

B

MEAMAASTSLPDPGDFDRNVPRICGVCGDRATGFHFNAMTCEGCKGFFRRSMKRKALFTCPFNGDCRIT
KDNRRHCQACRLKRCVDIGMMKEFILTDEEVQRKREMILKRKEEEALKDSLRPKLSEEQQRIIAILLDA
HHKTYDPTYSDFCQFRPPVRVNDGGGSHPSRPNSRHTPSFSGDSSSSCSDHCITSSDMMDSSSFSNLDL
SEEDSDDPSVTLELSQLSMLPHLADLVSYSIQKVIGFAKMIPGFRDLTSEDQIVLLKSSAIEVIMLRSN
ESFTMDDMSWTCGNQDYKYRVSDVTKAGHSLELIEPLIKFQVGLKKLNLHEEEHVLLMAICIVSPDRPG
VQDAALIEAIQDRLSNTLQTYIRCRHPPPGSHLLYAKMIQKLADLRSLNEEHSKQYRCLSFQPECSMKL
TPLVLEVFGNEISXLGQPVAVPGWGCSSRATCQARGWRLLSSPPHPVWGSAPPLPPPLSTQPILSPVQP
NPFPAGFSPVP

Figure S2. Anti-VDR and anti-VDRx western blots of anti-GFP immunoprecipitates prepared from HEK-293T cells transfected with GFP-VDR-TGA (TGA), GFP-VDR-TGG (TGG) or GFP-VDR-TAATAA (TAATAA) as indicated.

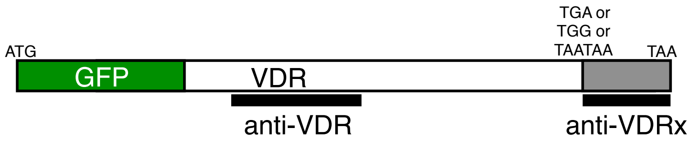

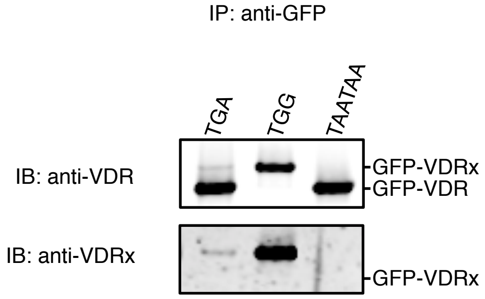

Figure S3. Fluorescence microscopy of live HeLa cells imaged 24 hr after transfection with the indicated GFP constructs. Transfectants were imaged before (Resting) and after (Stimulation) the addition of 10^{-7} M calcitriol (D3) for 10 min. Nuclei are shown in red (Hoecsht 33342 staining - HXT). Scale bar is in μm.

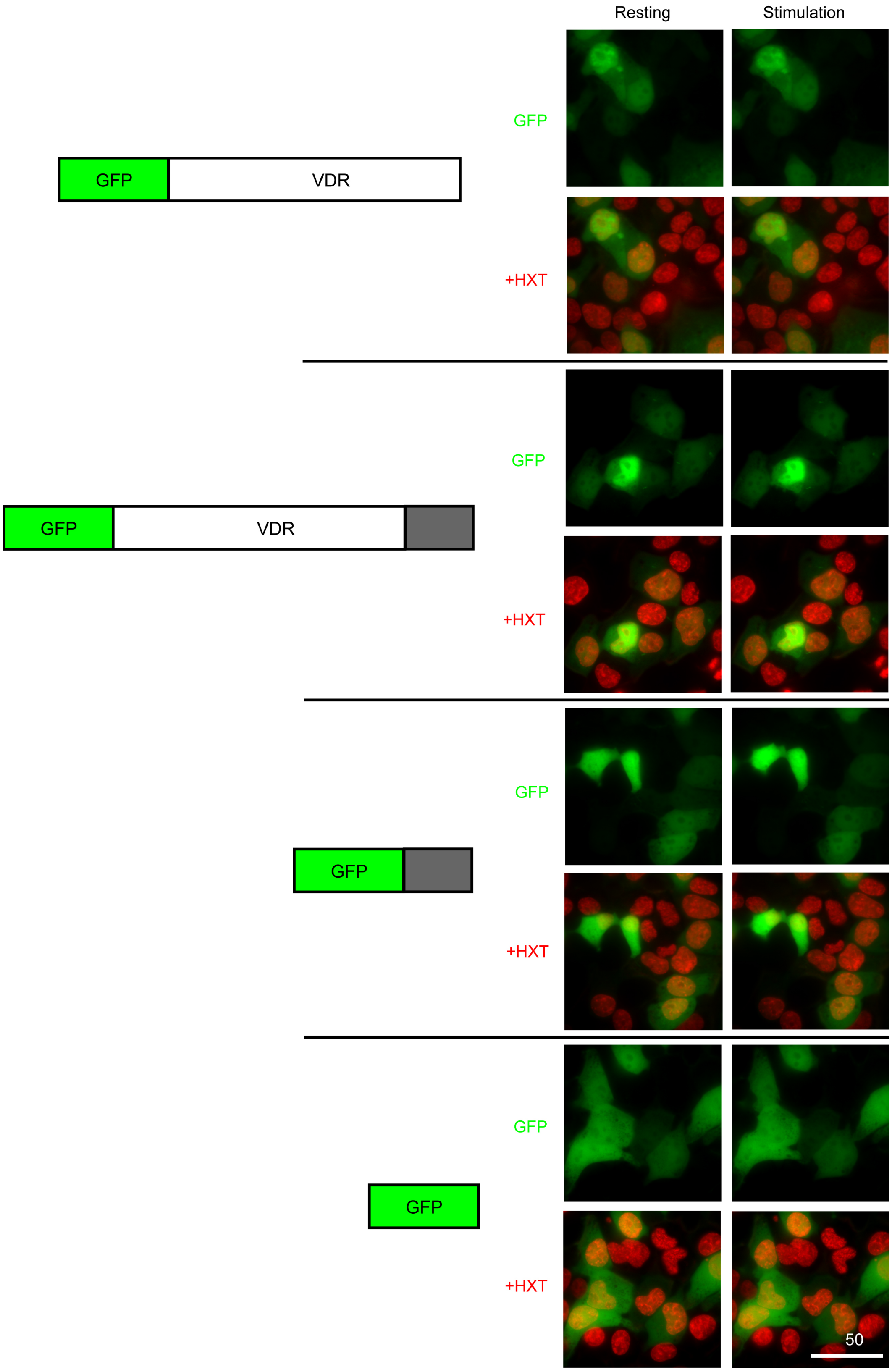

Supplement: Supporting Information [file supp_M117.818526_2017-818526_Supplementary.pdf]
